# Thiosemicarbazone *p*-Substituted Acetophenone Derivatives Promote the Loss of Mitochondrial Δ**ψ**, GSH Depletion, and Death in K562 Cells

**DOI:** 10.1155/2015/394367

**Published:** 2015-05-05

**Authors:** Felipe S. Pessoto, Cesar H. Yokomizo, Tatiana Prieto, Cleverton S. Fernandes, Alan P. Silva, Carlos R. Kaiser, Ernani A. Basso, Iseli L. Nantes

**Affiliations:** ^1^NanoBioMAv, Centro de Ciências Naturais e Humanas, Universidade Federal do ABC, Avenida dos Estados 5001, Bairro Bangu, 09210-580 Santo André, SP, Brazil; ^2^Departamento de Biologia Molecular, Universidade Federal de São Paulo, Rua 3 de Maio 100, Vila Clementino, 04044-020 São Paulo, SP, Brazil; ^3^Departamento de Química, Universidade Estadual de Maringá, Avenida Colombo 5790, 87020-900 Maringá, PR, Brazil; ^4^Instituto de Química-Universidade Federal do Rio de Janeiro (IQ-UFRJ), Edifício do Centro de Tecnologia, Bloco A, Cidade Universitária, 21.941-909 Rio de Janeiro, RJ, Brazil

## Abstract

A series of thiosemicarbazone (TSC) *p*-substituted acetophenone derivatives were synthesized and chemically characterized. The *p*-substituents appended to the phenyl group of the TSC structures were hydrogen, fluor, chlorine, methyl, and nitro, producing compounds named TSC-H, TSC-F, TSC-Cl, TSC-Me, and TSC-NO_2_, respectively. The TSC compounds were evaluated for their capacity to induce mitochondrial permeability, to deplete mitochondrial thiol content, and to promote cell death in the K562 cell lineage using flow cytometry and fluorescence microscopy. TSC-H, TSC-F, and TSC-Cl exhibited a bell-shaped dose-response curve for the induction of apoptosis in K562 cells due to the change from apoptosis to necrosis as the principal mechanism of cell death at the highest tested doses. TSC-Me and TSC-NO_2_ exhibited a typical dose-response profile, with a half maximal effective concentration of approximately 10 *µ*M for cell death. Cell death was also evaluated using the 3-(4,5-dimethylthiazol-2-yl)-2,5-diphenyltetrazolium bromide (MTT) assay, which revealed lower toxicity of these compounds for peripheral blood mononuclear cells than for K562 cells. The possible mechanisms leading to cell death are discussed based on the observed effects of the new TSC compounds on the cellular thiol content and on mitochondrial bioenergetics.

## 1. Introduction

**Figure 1:**
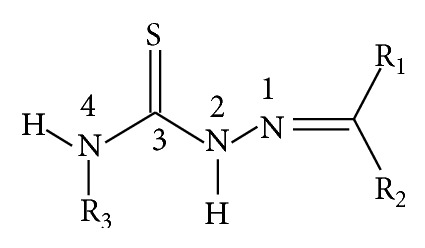
General structure of thiosemicarbazones with C=N-NH-CS-NH moiety; R1, R2, and R3 = alkyl or aryl groups.

Thiosemicarbazones (TSCs) are compounds that share the C=N-NH-CS-NH moiety, as shown in Figure 1.

**Scheme 1:**
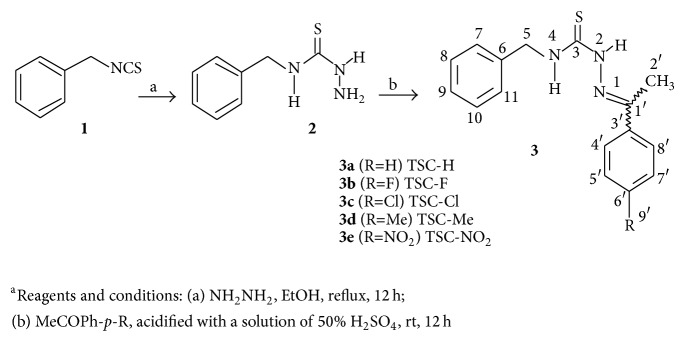
Synthesis of thiosemicarbazones.

The possibility of a puzzle combination of different groups at the variable positions 1, 2, and 4 makes this class of organic compounds extremely versatile for use as therapeutic agents for a variety of pathological conditions. Therefore, TSCs and their metal complexes [1, 2] possess potent antitumoral [3–9], antiviral [2, 5, 9–11], antibacterial [3, 5, 10, 12], and antiparasitic [2, 5, 11–14] properties. Anticonvulsant and neurotropic effects are also described in the literature [13, 15]. In some cases, these compounds are used clinically, such as the important chemotherapeutic anticancer agent 3-aminopyridine-2-carboxaldehyde thiosemicarbazone (i.e., Triapine) and its derivatives [2, 14–17]. Thus, for each specific therapeutic application, a unique structure is appropriated. In the case of the antifungal and antitumor activities of TSCs, the capacity to complex metal increases drug efficacy. The transition metal complex of TSCs can redox cycle, leading to the generation of free radical species; this process is an important contributing factor for the antitumor activity of these compounds [18]. Examples of TSC chelators with antitumor activity are the dipyridyl thiosemicarbazone (i.e., di-2-pyridyl ketone thiosemicarbazone (DpT)) and 2-benzoylpyridine thiosemicarbazone (BpT) classes. In addition to this nonspecific chelator effect, a specific activity has been associated with the antitumor activity of TSCs: the inhibition of ribonucleotide reductase, which is an enzyme that is required for the de novo synthesis of deoxyribonucleotides and DNA replication and repair [19]. 3-AP is a well-known example of this class of TSC compounds. 3-AP has also been suggested to act as a neuroprotectant during the treatment of neurodegenerative diseases [20–22]. Regardless of the mechanisms responsible for the diverse therapeutic effects of TSCs, these compounds are dependent on drug transfer through the plasma membrane. Therefore, the hydrophobic R groups and bulky substituent at the* p*-position of the rings are important for cytotoxicity. In the present study, we present a series of TSCs with benzyl as the R3 group,* p*-substituted phenyl as the R2 group, and methyl as the R1 group. These groups endow the molecules with a high hydrophobicity and favor transport through membranes. Furthermore, the R2 group was appended with different substituents to confer different properties to the molecules (Scheme 1). These TSC compounds were tested on K562 and peripheral blood mononuclear (PBMN) cells to determine their capacity to induce cell death and identify their effects on mitochondrial bioenergetics and thiol redox balance.

## 2. Materials and Methods

### 2.1. General

Chromatography was performed on silica gel Merck 230–400 mesh ASTM. All melting points were determined using a Microquimica model MQAPF-301 apparatus. The high-resolution electron spray ionization mass spectrometry (HRESIMS) analyses were performed on a QTOF Micro (Waters, Manchester, UK) mass spectrometer equipped with an ESI source. Proton nuclear magnetic resonance (1H NMR) spectra were recorded using CDCl_3_ as a solvent at ambient temperature using a Varian Mercury Plus (300 MHz) with TMS as an internal standard. The chemical shifts (*δ*) are given in parts per million relative to TMS. Carbon-13 nuclear magnetic resonance (13C NMR) spectra were recorded at 75.5 MHz with the same internal standard.

### 2.2. Synthesis

#### 2.2.1. N(4)-Benzyl-thiosemicarbazide (**2**)

To a solution of** 1** (5.00 g, 33.5 mmol) in ethanol (EtOH) (15 mL) was added hydrazine (1.07 g, 33.5 mmol). The reaction mixture was stirred at 90°C for 12 h. The resulting solid was collected by filtration, rinsed with cold EtOH, and recrystallized from ethyl acetate/methanol (EtOAc)/MeOH (2 : 1) to afford** 2** (5.80 g, 95%) as white crystals. ^1^H NMR (300 MHz, dimethyl sulfoxide-D6) *δ* 8.76 (br s, 1H, NH), 8.30 (br t,* J* = 6.3 Hz, 1H, NHCH_2_), 7.33–7.19 (m, 5H, Ar H), 4.71 (d,* J* = 6.3 Hz, 2H, NHCH_2_), 4.52 (br s, 2H, NH_2_); ^13^C NMR (75 MHz, dimethyl sulfoxide-D6) *δ* 182.6, 139.1, 128.0, 127.3, 126.6, 46.9. NMR data are in agreement with literature [5, 7].

#### 2.2.2. General Procedure for the Synthesis and Characterization of Compound** 3a–3e** (Scheme 1)

To a solution of 2 (0.30 g, 1.65 mmol) in ethanol was added, with stirring, a solution of ketone (1.65 mmol) and drops of an aqueous solution of sulfuric acid (50%). The mixture was stirred at room temperature for 12 h and then was concentrated in vacuum. The residue was partitioned between CHCl_3_ and 10% NaHCO_3_ aqueous solution and purified by silica gel column chromatography to afford** 3a–3e** [5, 7].

#### 2.2.3. N(1)-Methylphenyl Ketone-N(4)-benzyl-thiosemicarbazone-**3a** (TSC-H)

Starting from** 2** and methylphenyl ketone, the procedures summarized above provided the title compound in 61% yield (0.28 g) as white crystals after purification by column chromatography (silica gel, CHCl_3_/EtOAc, 100 : 0 to 2 : 1). ^1^H NMR (300 MHz, CDCl_3_) *δ* 8.76 (br s, 1H, NH), 7.92 (br t,* J* = 6 Hz, 1H, NHCH_2_), 7.66–7.63 (m, 2H, Ar H), 7.40–7.26 (m, 8H, Ar H), 4.98 (d,* J* = 6 Hz, 2H, NHCH_2_), 2.27 (s, 3H, CH_3_); ^13^C NMR (75 MHz, CDCl_3_) *δ* 178.6 (C3), 147.2 (C1′), 137.7 (C3′), 137.5 (C6), 129.9 (C5′ and C7′), 128.9 (C8 and C10), 128.7 (C9), 128.0 (C7 and C11), 126.5 (C4′ and C8′), 48.6 (C5), 13.8 (C2′). mp 157.4−159.7°C. HRMS (M + H^+^): 284.1218; C_16_H_18_N_3_S^+^ requires 284.1216.

#### 2.2.4. N(1)-Methyl-para-fluorophenylketone-N(4)-benzyl-thiosemicarbazone-**3b** (TSC-F)

Starting from** 2** and methyl-*para*-fluorophenylketone, the procedures summarized above provided the title compound in 54% yield (0.27 g) as a white solid after purification by column chromatography (silica gel, CHCl_3_/EtOAc, 100 : 0 to 2 : 1). ^1^H NMR (300 MHz, CDCl_3_) *δ* 8.82 (br s, 1H, NH), 7.88 (br t,* J* = 5.7 Hz, 1H, NHCH_2_), 7.64 (dd,* J* = 9.0 and* J*H-F = 5.1 Hz, 2H, Ar H), 7.40–7.26 (m, 5H, Ar H), 7.04 (dd,* J* = 9.0 and* J*H-F = 9.0 Hz, 2H, Ar H), 4.98 (d,* J* = 5.7 Hz, 2H, NHCH_2_), 2.26 (s, 3H, CH_3_); ^13^C NMR (75 MHz, CDCl_3_) *δ* 178.5 (C3), 163.8 (d,* J*C-F = 249.2 Hz, C6′), 146.3 (C1′), 137.7 (C6), 133.6 (d,* J*C-F = 3,8 Hz, C3′), 128.9 (C8, C9 and C10), 128.4 (d,* J*C-F = 8,3 Hz, C4′ and C8′), 127.8 (C7 and C11), 115.7 (d,* J*C-F = 21.7 Hz, C5′ and C7′), 48.5 (C5), 13.9 (C2′). mp 111.9−114.5°C. HRMS (M + H^+^): 302.1120; C_16_H_17_FN_3_S^+^ requires 302.1122.

#### 2.2.5. N(1)-Methyl-para-chlorophenylketone-N(4)-benzyl-thiosemicarbazone-**3c** (TSC-Cl)

Starting from** 2** and methyl-*para*-chlorophenylketone, the procedures summarized above provided the title compound in 47% yield (0.25 g) as a white solid after purification by column chromatography (silica gel, CHCl_3_/EtOAc, 100 : 0 to 2 : 1). ^1^H NMR (300 MHz, CDCl_3_) *δ* 8.73 (br s, 1H, NH), 7.86 (br t,* J* = 6 Hz, 1H, NHCH_2_), 7.58 (d,* J* = 8.7 Hz, 2H, Ar H), 7.40–7.28 (m, 7H, Ar H), 4.98 (d,* J* = 6 Hz, 2H, NHCH_2_), 2.26 (s, 3H, CH_3_); ^13^C NMR (75 MHz, CDCl_3_) *δ* 178.6 (C3), 146.0 (C1′), 137.6 (C6), 136.0 (C3′), 135.9 (C6′), 129.0 (C5′ and C7′), 128.0 (C8, C9 and C10), 127.9 (C7 and C11), 127.8 (C4′ and C8′), 48.7 (C5), 13.7 (C2′). mp 168.5–170.5°C. HRMS (M + H^+^): 318.0819; C_16_H_17_ClN_3_S^+^ requires 318.0826.

#### 2.2.6. N(1)-Methyl-para-methylphenylketone-N(4)-benzyl-thiosemicarbazone-**3d** (TSC-Me)

Starting from** 2** and methyl-*para*-methylphenylketone, the procedures summarized above provided the title compound in 45% yield (0.22 g) as white crystals after purification by column chromatography (silica gel, CHCl_3_/EtOAc, 100 : 0 to 2 : 1). ^1^H NMR (300 MHz, CDCl_3_) *δ* 8.82 (br s, 1H, NH), 7.93 (br t,* J* = 5.7 Hz, 1H, NHCH_2_), 7.54 (d,* J* = 8.1 Hz, 2H, Ar H), 7.40–7.25 (m, 5H, Ar H), 7.16 (d,* J* = 8.1 Hz, 2H, Ar H), 4.97 (d,* J* = 5.7 Hz, 2H, NHCH_2_), 2.34 (s, 3H, Ar-CH_3_), 2.23 (s, 3H, CH_3_); ^13^C NMR (75 MHz, CDCl_3_) *δ* 178.3 (C3), 147.4 (C1′), 140.0 (C6′), 137.7 (C6), 134.6 (C3′), 129.3 (C5′ and C7′), 128.8 (C8 and C10), 127.7 (C7 and C11), 127.6 (C9), 126.3 (C4′ and C8′), 48.4 (C5), 21.4 (C9′), 13.7 (C2′). mp 160.3−162.2°C. HRMS (M + H^+^): 298.1369; C_17_H_20_N_3_S^+^ requires 298.1372.

#### 2.2.7. N(1)-Methyl-para-nitrophenylketone-N(4)-benzyl-thiosemicarbazone-**3e** (TSC-NO_2_)

Starting from** 2** and methyl-*para*-nitrophenylketone, the procedures summarized above provided the title compound in 26% yield (0.14 g) as yellow crystals after purification by column chromatography (silica gel, CHCl_3_/EtOAc, 100 : 0 to 2 : 1). ^1^H NMR (300 MHz, CDCl_3_) *δ* 8.80 (br s, 1H, NH), 8.22 (d,* J* = 9.3 Hz, 2H, Ar H), 7.85 (br t,* J* = 5.7 Hz, 1H, NHCH_2_), 7.80 (d,* J* = 9.3 Hz, 2H, Ar H), 7.40–7.29 (m, 5H, Ar H), 5.00 (d,* J* = 5.7 Hz, 2H, NHCH_2_), 2.33 (s, 3H, CH_3_); ^13^C NMR (75 MHz, CDCl_3_) *δ* 178.7 (C3), 148.2 (C6′), 144.3 (C1′), 143.4 (C3′), 137.4 (C6), 129.1 (C8 and C10), 128.1 (C9), 128.0 (C7 and C11), 127.2 (C5′ and C7′), 124.0 (C4′ and C8′), 48.8 (C5), 13.7 (C2'). mp 156.8–158.0°C. HRMS (M + H^+^): 329.1071; C_16_H_17_N_4_O_2_S^+^ requires 329.1067.

### 2.3. HRESIMS Analyses

The high-resolution mass spectrometry analyses were performed on a QTOF Micro (Waters, Manchester, UK) mass spectrometer equipped with an ESI source. The analyses were recorded between* m/z* 90 and 1000 in positive ion mode, and the mass spectrometer parameters were as follows: the nebulization gas was set to 500 L/h at 140°C, the cone gas set to 50 L/h, and the source temperature set to 100°C. The capillary voltage was set to 4.5 kV and cone voltage set to 30 V. The QTOF acquisition rate was set to 1.0 s, with a 0.4 s interscan delay and the data processed on the MassLynx 4.0 software (Waters, Manchester, UK). Analytes were acquired using the LockSpray and phosphoric acid (0.1% in acetonitrile/water 1 : 1) as external and internal standard to ensure accuracy mass. The sample solutions (0.5 mg/mL) were prepared in acetonitrile with addition of 20 *μ*L of formic acid. The analyses were carried out by direct infusion using an Alliance HT 2795 HPLC system (Waters, Manchester, UK) at flow ratio of 150 *μ*L/min of acetonitrile/water 1 : 1 and injection volume of 10 *μ*L. The obtained results are showed in Table 1. HRESIMS* m/z* 284.1218 [M + H]^+^ (Calcd for C_16_H_18_N_3_S^+^: 284.1216); HRESIMS* m/z* 302.1120 [M + H]^+^ (Calcd for C_16_H_17_FN_3_S^+^: 302.1122); HRESIMS* m/z* 318.0819 [M + H]^+^ (Calcd for C_16_H_17_ClN_3_S^+^: 318.0826); HRESIMS* m/z* 298.1369 [M + H]^+^ (Calcd for C_17_H_20_N_3_S^+^: 298.1372); HRESIMS* m/z* 329.1071 [M + H]^+^ (Calcd for C_16_H_17_N_4_O_2_S^+^: 329.1067). The mass values are presented in Table 1.

### 2.4. Determination of Total Thiol Content

After 15 min incubation under swelling conditions, mitochondria were treated with trichloroacetic acid (5% final concentration) and centrifuged at 4500 g for 10 min. The pellet was suspended with 1 mL of 0.5 M potassium phosphate buffer, pH 7.6, and, after addition of 0.1 mM DTNB, absorbance was determined at 412 nm. The amount of accessible sulfhydryl groups was calculated by measuring the TNB released using a molar extinction coefficient of 13.600 M^−1^ cm^−1^ [23].

### 2.5. Determination of K562 Reduced Glutathione Content

After 15 min incubation under swelling conditions, mitochondria suspension was treated with 0.5 mL of 13% trichloroacetic acid and centrifuged at 900 g for 3 min. Aliquots (100 *μ*L) of the supernatant were mixed with 2 mL of 100 mM NaH_2_PO_4_ buffer, pH 8.0, containing 5 mM EGTA. One hundred microliters of* o*-phthalaldehyde (1 mg/mL) was added, and the fluorescence was measured 15 min later using the 350/420 nm excitation/emission wavelength pair in a F-2500 fluorescence spectrophotometer (Hitachi, Ltd., Tokyo, Japan) [24].

### 2.6. Isolation of Peripheral Blood Mononuclear (PBMN) Cells

The mononuclear lymphocytes were isolated by density gradient centrifugation. The blood volume was firstly 4-fold diluted with Roswell Park Memorial Institute medium (RPMI) 2 mM EDTA; 7 mL from diluted blood was carefully layered over 3 mL of Histopaque-1077 (Sigma); centrifuged at 400 ×g for 40 min, the mononuclear cloud layer was gently aspirated and diluted in 50 mL of RPMI; centrifuged at 300 ×g for 15 min, the pellet was suspended in 40 mL of RPMI and centrifuged at 200 ×g for 10 min (twice); finally the cells were suspended in an appropriated volume for MTT assay.

### 2.7. Cell Culture and Cell Viability Assay

K562 and mononuclear cells were cultured in RPMI 1640 medium supplemented with 10% fetal bovine serum (FBS), 100 U/mL penicillin, and 100 mg/mL streptomycin (Invitrogen, Carlsbad, CA, USA) at 37°C/5% CO_2_ (Sanyo MCO-20AIC, Japan). For viability experiments, cells were maintained in grown media at 2 × 10^5^ cells/well for both cells lines and they were added to 96-well microplates (0.2 mL final volume) in the presence of different concentrations (1 *μ*M–2 mM) of TSC-Me and TSC-NO_2_ and incubated for 3 and 6 h. After incubation time, 0.25 mg/mL MTT (3-(4,5-dimethylthiazol-2-yl)-2,5-diphenyl-2H-tetrazolium bromide) was added and after 4 h, the cells and MTT crystals were solubilized in 0.1 mL of 10% SDS/0.01 M HCl overnight. The final absorbance assay was read at 630 nm (Biochrom Asys Expert Plus, Cambridge, UK). Cell viability was determined relative to the control, performed in the absence of TSC compounds, and considered as 100% reference. Results are presented as the mean ± S.D. of three independent experiments.

### 2.8. Annexin Fluorescein Isothiocyanate/7-Amino-actinomycin D (V-FITC/7-ADD) Double-Staining and Flow Cytometry Analysis

After treatment with TSC for 6 and 24 h as described above, K562 cells were harvested, washed with cold PBS, and suspended in binding buffer (0.01 M HEPES, pH 7.4, 0.14 M NaCl, and 2.5 mM CaCl_2_) at a concentration of 1 × 10^6^ cells/mL. The suspensions were transferred to 5-mL tubes and 5 *μ*L annexin V-FITC and 5 *μ*g/mL 7-ADD were added. The cells were incubated at 25°C for 20 minutes and after the addition of 0.3 mL of binding buffer, the analysis was performed in a flux cytometer Beckman Coulter, model Lab Cell Quanta SC MPL. Control cells were treated only with the medium. Data were present as media ± S.D. of the triplicates.

### 2.9. Cytomorphology

Microscopy images of the cells were obtained using a Widefield Leica DMI 6000B microscope (Leica Microsystems, Germany) with an objective HCX PL APO 40x/0.85 coupled to an ultra-fast digital camera Leica DFC365 FX (Leica Microsystems, Germany). The nuclei in the same visual field were stained with 4′,6-diamidino-2-phenylindole (DAPI) (10 *μ*g/mL) and energized mitochondria were stained by the retention of the dye 3,3′-dihexyloxacarbocyanine iodide (DiOC_6_(3)) incubated at 37°C for 30 min.

## 3. Results and Discussion

### 3.1. Synthesis and Chemical Characterization of the TSCs

The five novel compounds TSC-Cl, TSC-Me, TSC-F, TSC-H, and TSC-NO_2_ were prepared using the procedure described in Scheme 1. The synthesis of TSCs** 3a–3e** was based on benzyl-thiosemicarbazide (**2**), which was obtained by reacting hydrazine with benzyl-isothiocyanate (**1**) in ethanol at 90°C. After isolation, the ethanol solution of compound** 2** and the acetophenone derivatives were stirred at room temperature in the presence of drops of 50% sulfuric acid until the complete consumption of compound** 2** occurred. TSCs** 3a–3e** (Scheme 1) were obtained with a good yield after purification of the resulting reaction mixture by elution through a silica gel chromatography column. Compounds** 3a–3e** were characterized via the combined use of HRESIMS, 1D nuclear magnetic resonance (NMR) and 2D NMR, and subsequent comparison with data available in the literature [25–27]. The 1D NMR spectra of general structure** 3** exhibited signals at *δ*_H_/*δ*_C_ 2.23–2.33 (s, 3H)/13.7–13.9 from the methyl group; 4.97–5.00 (d, 2H)/48.4–48.8 from the methylene group; 7.85–7.04/165.4–115.7 from the aromatic rings; and *δ*_H_ 8.73–8.82 and 7.86–8.22 from the NH groups; in addition, the signals *δ*_C_ 143.3–147.4 and 178.3–178.7 originated from the C=N and C=S groups, respectively.

The log⁡⁡*P* magnitude was calculated using Advanced Chemistry Development, Inc. (ACD/Labs) ChemSketch software, Toronto, ON, Canada, and the following values were obtained: 4.19 ± 0.89, 3.89 ± 0.59, 3.65 ± 0.62, 3.43 ± 0.59, and 3.39 ± 0.60. The high values of log⁡⁡*P* indicated that these compounds are highly hydrophobic and have a high affinity for the lipid bilayers of biological membranes. These compounds were then tested for their capacity to promote cell death in association with mitochondrial permeability and depletion of thiol content.

### 3.2. Effect of TSCs on Mitochondrial Permeability and the Total Content of Protein Thiol Groups

**Figure 2:**
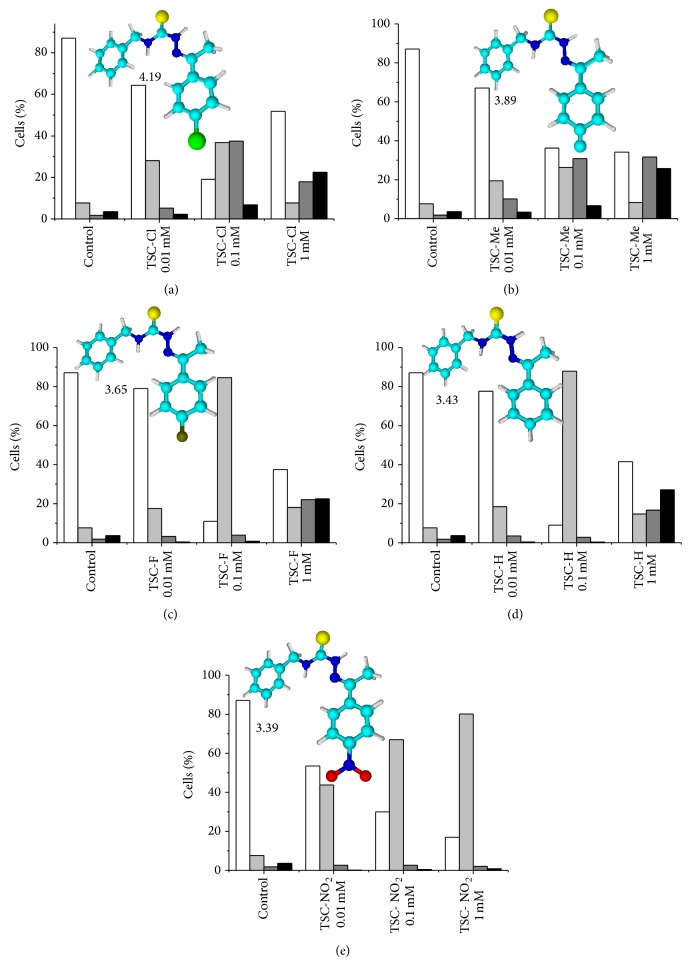
Cell death, apoptosis, and necrosis were triggered in K562 cells by TSC compounds in a dose-dependent manner. K562 cells were incubated with 0.01, 0.1, and 1.0 mmol/L of (a) TSC-Cl, (b) TSC-Me, (c) TSC-F, (d) TSC-H, and (e) TSC-NO_2_ for 6 h. Apoptosis and necrosis were evaluated via flow cytometric analysis of phosphatidylserine externalization (i.e., annexin V binding) and cell membrane integrity (i.e., 7-AAD staining). White columns correspond to the percentage of the viable cell population, light gray columns correspond to early apoptotic cells, gray columns correspond to late apoptotic cells, and black columns correspond to necrotic cells. The insets show the structures of the compounds with the corresponding calculated log⁡⁡*P* values.

The TSC compounds were tested in cultured K562 cells at the concentrations of 10, 100, and 1000 *μ*M. The cells were incubated with TSC compounds, and the progression of apoptosis was analyzed using a flow cytometer with the annexin V-FITC/7-AAD kit. Cells lose membrane phospholipid asymmetry during the early step of apoptosis, and phosphatidylserine (PS), which is a negatively charged phospholipid that is located in the inner surface of the plasma membrane, appears on the external cell surface. During this step, cell membrane integrity is preserved. Fluorescein-labeled annexin V binds preferentially to PS and stains dying cells before they hydrolyze their DNA and lose their morphology; thus, annexin V staining characterizes cells during early apoptosis. As apoptosis progresses, the cells are stained by the DNA-specific viability dye 7-AAD [28–32]. Thus, in the presence of both annexin V and 7-AAD, it is possible to distinguish different cell populations. Cells undergoing early apoptosis are stained only with annexin V, late apoptotic cells are stained with both annexin V and 7-AAD, necrotic cells are stained only with 7-AAD, and live cells are not stained by any dye. Figures 2(a), 2(b), 2(c), 2(d), and 2(e) present the results obtained using TSC-Cl, TSC-Me TSC-F, TSC-H, and TSC-NO_2_, respectively. The insets show the 3D structures of the respective TSCs. The compounds are arranged from (a) to (e) in decreasing order of the log⁡⁡*P* magnitude, which was calculated using ACD/lab ChemSketch software. All probed TSC compounds were able to trigger cell death, but these compounds had unique profiles.

Figure 2 reveals three different patterns of dose-dependent cell death that were promoted by TSC compounds. The compounds TSC-Cl, TSC-Me, TSC-F, and TSC-H exhibited bell-shaped profiles of their dose-response effects. TSC-Cl and TSC-Me had the highest calculated log⁡⁡*P* values and exhibited very similar dose-response curves. After a 6 h incubation with 100 *μ*M TSC-Cl, ~80% of the K562 cells were in the death process, with equal contributions from cells in early apoptosis and cells in late apoptosis. K562 cells that were incubated with 1 mM TSC-Cl remained ~50% viable, and necrosis was the predominant mechanism of cell death. This peculiar dose-response effect with a switch from apoptosis to necrosis could result from the aggregation of the TSC molecules at high concentrations. This aggregation could result in the formation of some particles with a lower availability for endocytosis. However, the eventual endocytosis of aggregated particles by some cells could result in a high individual dosage, leading to necrosis. TSC-Me exhibited a dose-response profile similar to that of TSC-Cl, but the percentage of viable cells decreased with increasing TSC-Me concentrations, with saturation at 1 mM. For TSC-Me, the switch from apoptosis to necrosis was not observed, although a significant increase in the cell population undergoing necrosis was observed at a dose of 100 *μ*M.

The dose-response data obtained for TSC-H and TSC-F, which had intermediate calculated log⁡⁡*P* values, were nearly identical. For those compounds, early apoptosis was the predominant event observed in K562 cells after a 6 h incubation in the presence of 10 or 100 *μ*M TSC. At the latter concentration, more than 80% of the cells were undergoing early apoptosis after a 6 h incubation. However, after a 6 h incubation with 1 mM TSC-H or TSC-F, 40% of the K562 cells remained viable and the remaining 60% of the cells, which were detected in a death process, were in early apoptosis, apoptosis, or necrosis, which was the most abundant population when the cells were treated with TSC-H. The cells treated with 1 mM TSC-F were undergoing apoptosis and necrosis, with similar percentages.

K562 cells that were incubated with different doses of TSC-NO_2_, which had the lowest calculated log⁡⁡*P* value, exhibited early apoptosis as the predominant mechanism of cell death at all of the tested doses; the percentage of viable cells decreased in a dose-dependent manner. For TSC-NO_2_, the EC50 for apoptosis was ~10 *µ*M, which is lower than or in the range of other TSC compounds reported in the literature [33–36].

To investigate the possible involvement of mitochondrial bioenergetics in the induction of apoptosis by the investigated TSC compounds, the mitochondrial membrane potential (Δ*ψ*_*m*_) was assessed using fluorescence microscopy of cells labeled with the dye DiOC_6_(3). DiOC_6_(3) is a cell-permeant, green-fluorescent, lipophilic dye, which selectively stains the energized mitochondria of live cells at low concentrations [37–42].

**Figure 3:**
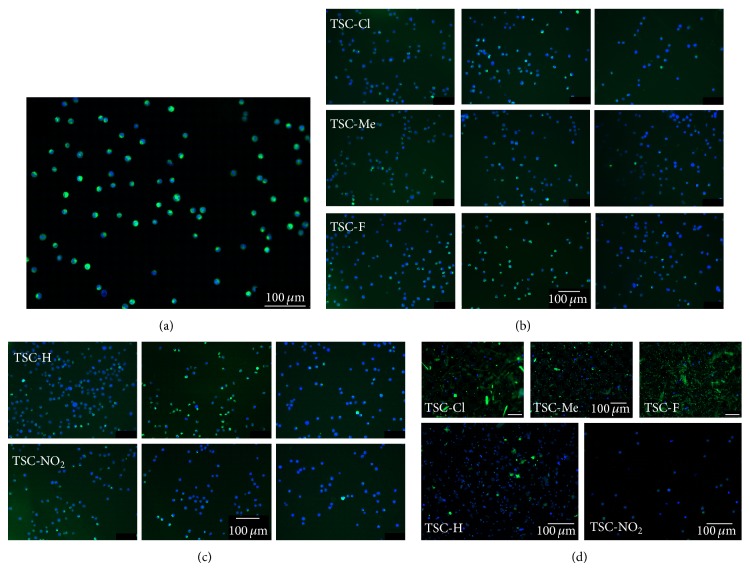
Effect of TSC compounds on mitochondrial membrane potential and nuclear morphology after exposure to TSC compounds. Simultaneous measurements of Δ*ψ*_*m*_ (DiOC_6_(3), green fluorescence) and nuclear morphology (DAPI, blue fluorescence) were made in K562 cells after different incubation times in the absence (control) and in the presence of TSC compounds. Bleaching of the green fluorescence of DiOC_6_(3) indicates the disruption of Δ*ψ*_*m*_. Panel (a) shows an image from a control sample of K562 cells after 6 h incubation in the absence of TSC compounds. From left to center, panel (b) shows images of K562 cells after 3 h incubation in the presence of 0.01 or 0.1 mM of the indicated TSC compounds (i.e., TSC-Cl, TSC-Me, and TSC-F); the right side shows images of K562 cells after 1 h incubation in the presence of 1 mM of the same compounds. From left to center, panel (c) shows images of K562 cells after 3 h incubation in the presence of 0.01 or 0.1 mM of the indicated TSC compounds (i.e., TSC-H and TSC-NO_2_); the right side shows images of K562 cells after 1 h incubation in the presence of 1 mM of the same compounds. Panel (d) shows images of K562 cells after 6 h incubation in the presence of 1 mM of the indicated TSC compounds, that is, TSC-Cl, TSC-Me, TSC-F, TSC-H, and TSC-NO_2_.

**Figure 4:**
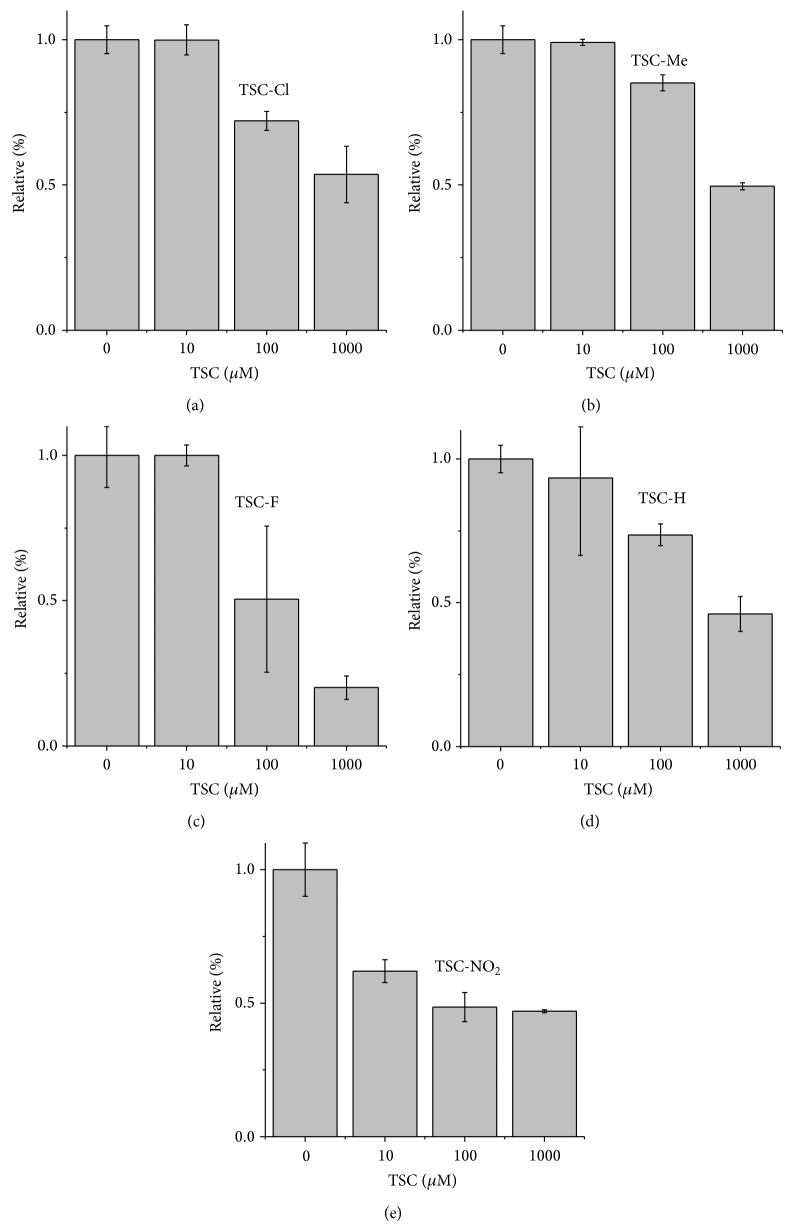
Depletion of GSH in K562 cells by the indicated TSC compounds. GSH levels in K562 cells were determined based on fluorescence after 15 min incubation with* o*-phthalaldehyde (1 mg/mL). The fluorescence measurements were determined using the 350/420-nm excitation/emission wavelength pair in an F-2500 fluorescence spectrophotometer, as described in Section 2.

Figure 3 shows the effects of TSC compounds on the Δ*ψ*_*m*_ and nuclear morphology of K562 cells. Panel (a) shows a representative image of a control sample of K562 cells after a 6 h incubation in the absence of TSC compounds. In the absence of TSC compounds, K562 cells appeared normal, with round and homogenous nuclei. Green fluorescence in the majority of cells indicates that the mitochondria are coupled. Panels (b) and (c) show that significant loss of Δ*ψ*_*m*_ was observed after a 3 h incubation in the presence of 0.01 and 0.1 mM of the studied TSC compounds, that is, TSC-Cl, TSC-Me, TSC-F TSC-H, and TSC-NO_2_. However at the concentration of 1 mM, 1 h of incubation was enough for a complete disruption of Δ*ψ*_*m*_ of K562 cells that were treated with the TSC compounds. Panel (d) shows images of K562 cells after a 6 h incubation in the presence of 1 mM of the TSC compounds. The images are consistent with the flow cytometry results. K562 cells treated with 1 mM concentrations of TSC-Cl, TSC-Me TSC-F, and TSC-H exhibited characteristics of necrosis, with nuclear fragmentation. Consistently, characteristics of necrosis were not detected for cells treated with TSC-NO_2_. The green fluorescence present in these conditions can be explained by the behavior of DiOC_6_(3). Once DiOC_6_(3) is detached by depolarized mitochondria, it binds to other biological membranes, such as membranes from fragmented organelles. It is not uncommon that the loss of Δ*ψ*_*m*_ is associated with the depletion of cellular thiol content [43]. Therefore, the membrane protein thiol and glutathione (GSH) content of K562 cells was analyzed after incubation with the TSC compounds (Figure 4).

**Figure 5:**
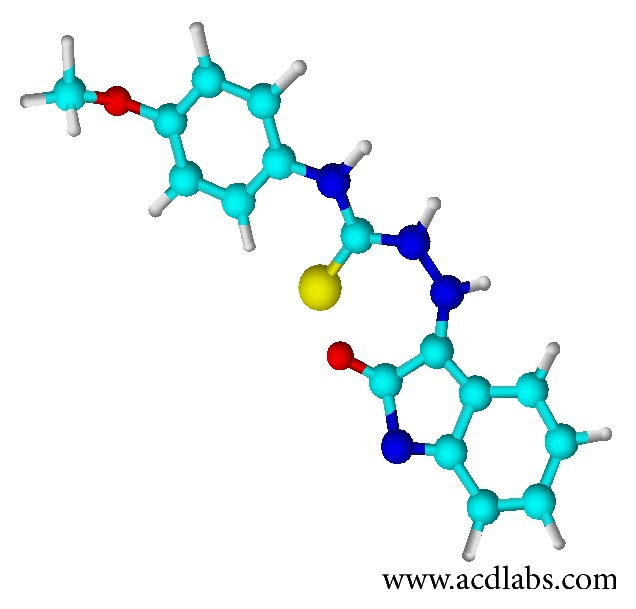
3D structure of the compound NSC73306.

The TSC compounds did not promote significant changes in the membrane protein thiol content (data not shown). However, a dose-dependent decrease in GSH content was observed for K562 cells treated with the TSC compounds. A direct correlation between the extension of apoptosis and the disruption of Δ*ψ*_*m*_ and the depletion of GSH was not observed for cells treated with TSC compounds. Interestingly, TSC-NO_2_ had peculiar and consistent effects on the GSH content and Δ*ψ*_*m*_ of K562 cells. TSC-NO_2_ was the only TSC compound that was able to promote cell death exclusively by apoptosis, with significant GSH depletion at low concentrations (10 *μ*M). The depletion of GSH by the TSC compounds studied here could be related to the activation of the ABCB1 transporter and quinone reductase 2 (QR2) activity. TSCs with a* para* substituted phenyl moiety, which are exemplified by the structure of 1-isatin-4-(40-methoxyphenyl)-3-thiosemicarbazone (NSC73306, Figure 5), have been reported to be a target for the ABCB1 transporter, whose mechanism involves GSH consumption. Differently, the activity of QR2 involves generation of hydroxyl radical [44].

**Scheme 2:**
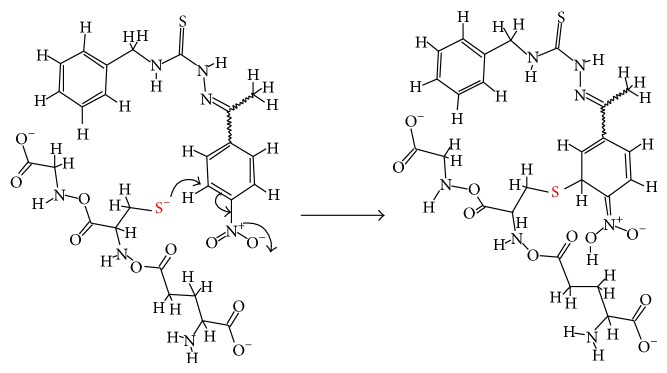
Mechanism of the reaction of the thiol group cysteine lateral chain with a nitrophenyl moiety (based on Tiwari et al., [45]).

A more plausible mechanism for the depletion of GSH that is promoted by TSC compounds, particularly TSC-NO_2_, is a nonenzymatic reaction with the peptide thiol groups. This putative mechanism is inspired by the mechanism proposed by Tiwari et al. [45] for the formation of nitroso-intermediates of nitroaromatic compounds with antituberculosis (TB) activity. Nitroaromatic compounds, such as 1,3-benzothiazin-4-ones (BTZs) and similar compounds, generate nitroso-intermediates in vivo that are able to cause suicide inhibition of decaprenylphosphoryl-*β*-D-ribose 2′ oxidase (DprE1). This enzyme inhibition causes the death of* Mycobacterium tuberculosis* because DprE1is responsible for the biosynthesis of arabinogalactan in the cell wall. According to computational studies, the authors demonstrated that the nonsubstituted aromatic carbons of nitroaromatic compounds are the most electron-deficient and the most susceptible to nucleophilic attack. Therefore, nucleophiles, such as thiolates of glutathione, might induce the nonenzymatic reduction of the nitro groups in the structure of these compounds (Scheme 2). This mechanism involves the addition of the nucleophiles at the nonsubstituted electron-deficient aromatic carbon that is present in these compounds.

The fate of the nitrocompound after the reaction with GSH might be the generation of NO^•^, which is consistent with the induction of cell death in cultured cells. Another possibility is the formation of a nitroso derivative that also exerts toxic effects on cells.

**Figure 6:**
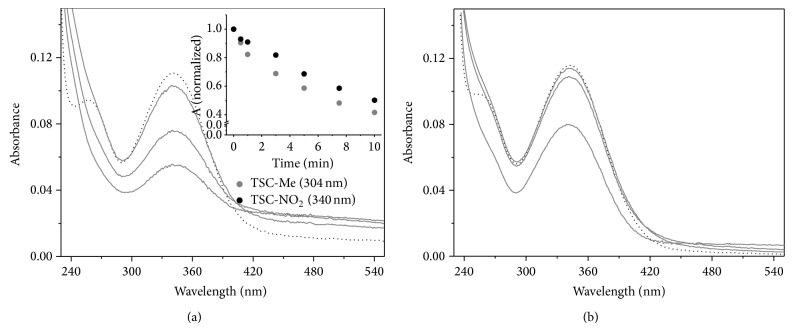
Reaction of TSC compounds with GSH. (a) Time-dependent bleaching of 10 *μ*M TSC-NO_2_ incubated with 20 *μ*M GSH in a nitrogen-equilibrated solution. (b) Time-dependent bleaching of 10 *μ*M TSC-NO_2_ incubated with 20 *μ*M GSH in an air-equilibrated solution. The inset of (a) shows the kinetics of TSC-Me (gray ball) and TSC-NO_2_ (black ball) bleaching at 304 and 340 nm, respectively, in nitrogen-equilibrated solutions.

To investigate the capacity of the TSC compounds to react nonenzymatically with the thiolate groups of proteins and peptides, two representative TSC compounds (i.e., TSC-Me and TSC-NO_2_) were incubated with glutathione and electronic absorbance spectral analyses of air- and nitrogen-equilibrated solutions were performed (Figures 6(a) and 6(b)). These TSC compounds were chosen based on the presence of an electron donor and an electron-withdrawing* p*-substituent in TSC-Me and TSC-NO_2_, respectively.

Figure 6(a) shows that TSC-NO_2_ and TSC-Me exhibited time-dependent bleaching after incubation with GSH in an N_2_-equilibrated solution.  Figure 6(b) shows that the bleaching of TSC-NO_2_ is mitigated by the presence of molecular oxygen. Similar results were obtained for TSC-Me in an air-equilibrated solution (data not shown). The occurrence of TSC bleaching is consistent with the loss of aromatic conjugation that is expected to occur if the reaction presented in Scheme 2 occurred. However, in the case of TSC-Me, the mechanism described for TSC-NO_2_ is not expected and the bleaching could be caused by the reduction of the TSC moiety, as described by Andrade and Temperini [46]. The exclusive capacity of TSC-NO_2_ to deplete GSH at low concentrations in association with the occurrence of cell death exclusively by apoptosis suggests that, for the nitro TSC, the mechanism described in Scheme 2 might play a role. The reduction of TSC produces thiocarbamide (thiourea) [47], whose toxic effects are well-known [48–50].

TSCs that possess a coordinating ring-*N* were previously reported to be efficient metal chelators due to their capacity to form a tridentate complex [51]. These TSC compounds exhibited high antiproliferative activity associated with a significant iron (Fe) chelation ability. This antiproliferative effect in association with iron chelation was corroborated by probing the effect of related TSCs that do not possess a coordinating ring-*N* (i.e., acetophenone thiosemicarbazone and acetophenone* N*,*N*-dimethylthiosemicarbazone). These compounds exhibited little activity. The antiproliferative effect of metal chelator TSCs results from Fe chelation and mobilization. The literature has also attributed this antiproliferative effect to the generation of free radicals via the redox cycling of Fe complexes. The TSC compounds investigated in the present study do not have a coordinating ring-*N* in their structures; thus, these compounds can form only a bidentate complex, as shown as an inset of Figure 6.

In addition, these compounds exhibit high hydrophobicity (log⁡⁡*P* > 3.0) that might contribute to the permeabilization of biological membranes. Therefore, synergistic effects of metal chelation, changes in membrane fluidity, and GSH depletion might contribute to cell death.

**Figure 7:**
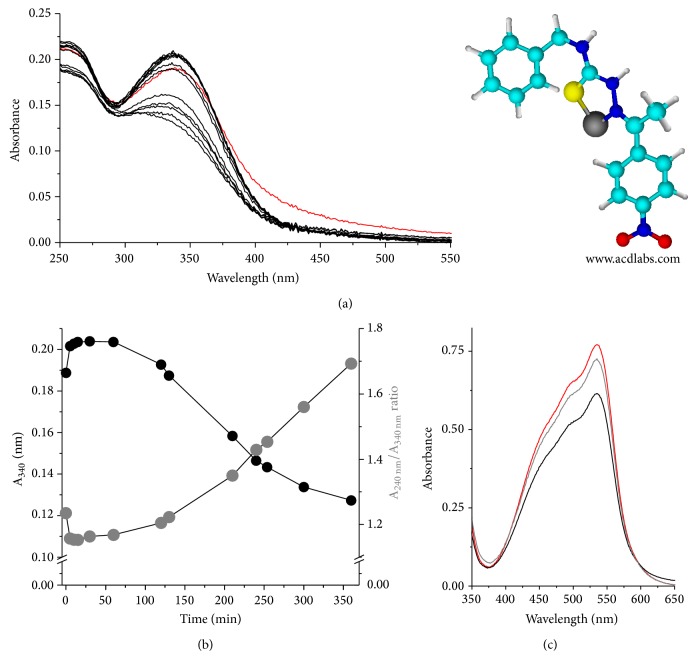
Spectral changes of TSC compounds associated with metal complexation. (a) Spectral changes of TSC-NO_2_ after the addition of ferrous sulfate. The red line corresponds to the initial spectrum before metal addition. The structure of TSC-NO_2_ complexed with Fe^2+^ is shown beside the spectrum. (b) A progressive decrease of the band that peaks at 340 nm and increase of the A_240_ nm/A_340_ nm ratio are associated with the disaggregation of TSC-NO_2_ and the formation of metal complexes. Panel (c), black line, shows the spectrum obtained when bathophenanthroline is added to the medium containing ferrous sulfate after 6 h incubation of with TSC-NO_2_. The red line corresponds to the spectrum of bathophenanthroline added to a fresh solution of ferrous sulfate, and the gray line corresponds to the control, that is, bathophenanthroline added to a solution of ferrous sulphate incubated for 6 h in the absence of TSC-NO_2_.

The capacity for metal chelation was tested for TSC-NO_2_ and TSC-Me. The incubation of TSC-NO_2_ with ferrous sulfate led to spectral changes for the compound (Figure 7(a)). Figure 7(a) demonstrates that immediately after the addition of a molar excess of ferrous sulfate (50 *μ*M), 15 *µ*M TSC-NO_2_ exhibited a narrowing of the band peaking at 340 nm in association with an increase in intensity. The narrowing and increased intensity of the 340 nm band are consistent with the conversion of aggregate forms of TSC-NO_2_ to the monomeric form. This possibility was reinforced by the titration of crescent concentrations of TSC-NO_2_ in solution, showing that increases in TSC-NO_2_ concentration led to a red shift and a broadening of the 340 nm band (data not shown). The disaggregation of TSC-NO_2_ molecules after ferrous sulfate addition is suggestive of the formation of metal complexes. After ~40 min of incubation of TSC-NO_2_ with ferrous sulfate, the compound exhibited a progressive reduction of the band that peaked at 340 nm in association with an increase in the A_240_ nm/A_340_ nm ratio, as shown in panel (b). These results are consistent with the reduction of TSC compounds by ferrous ions [46]. Consistent with the formation of a bidentate metal complex, the TSC compounds were unable to compete with bathophenanthroline. The spectrum of bathophenanthroline obtained immediately after its addition to a fresh solution of ferrous sulfate in the absence of TSC-NO_2_ (Figure 7(c), red line) presented the same intensity of that obtained in the presence of the TSC compound (not shown). However, the addition of bathophenanthroline to a ferrous sulfate solution incubated for 6 h with 15 *μ*M TSC-NO_2_ presented an absorbance intensity (Figure 7(c), black line) consistent with a reduction of the ferrous ion content (~15%) compared to the control which was incubated for 6 h in the absence of TSC-NO_2_ (Figure 7(c), gray line). A 15% decrease in ferrous ion content corresponds to the oxidation of ~7.5 *μ*M of ferrous sulfate and the reduction of an equal concentration of TSC-NO_2_. This calculation is consistent with the observed ~50% decrease in the TSC-NO_2_ (15 *μ*M) band that occurred after a 6 h incubation with ferrous sulfate. The bleaching of TSC-NO_2_ and TSC-Me was also observed in an air-equilibrated solution (data not shown), and after a 24 h incubation, both of the TSC compounds exhibited intense bleaching. These results suggest that complex redox processes can occur with TSC compounds in cells. The elucidation of these processes and the identification of the products are not the focus of the present study, and these topics will be investigated in the future.

**Figure 8:**
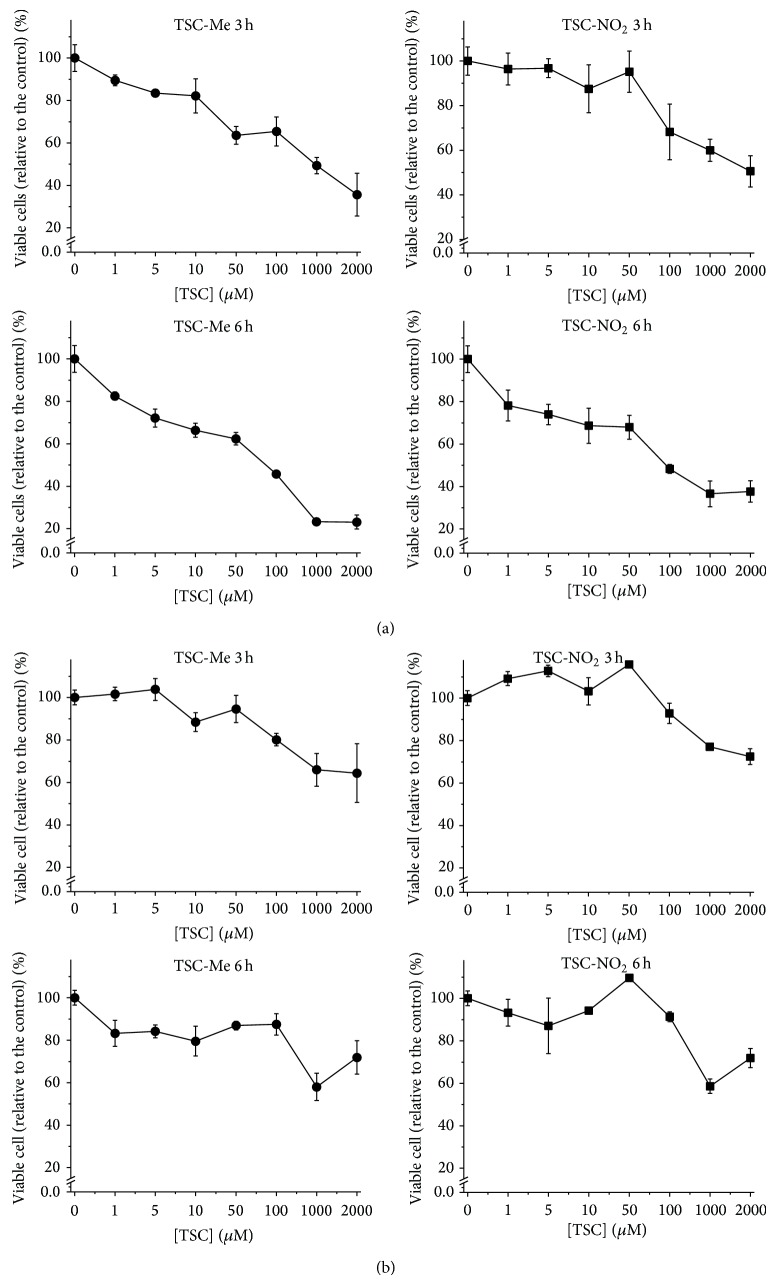
Viability of K562 and PBMN cells after 3 and 6 h of incubation with TSC-Me and TSC-NO_2_. (a) K562 and (b) PBMN cell viability was evaluated by the MTT reduction test. Cells (2 × 10^5^ per well) were incubated with different concentrations of drug for 3 and 6 h. After the incubation, MTT was added and the absorbance at 630 nm was measured. The percentage of viable cells after treatment with TSC-Me and TSC-NO_2_ is expressed relative to the control (absence of TSC compounds), which was considered as 100%. Results presented are means ± S.D. of three independent experiments.

Considering the capacity of TSC compounds to promote the death of K562 cells, it was important to investigate the comparative toxic effects of these compounds for peripheral blood mononuclear cells (PBMNs). A comparison of these toxic effects was performed for cells treated with TSC-Me and TSC-NO_2_ using MTT assays.  Figure 8 demonstrated that, in contrast to the observations made for K562 cells (i.e., ~25% viability), more than 50% of the PBMNs remained viable, even after a 6 h incubation with 2 mM TSC-NO_2_ and TSC-Me.

Taken together the present study demonstrated the efficiency of new TSC compounds to kill K562 cells with peculiarities for the nitrocompound TSC-NO_2_. Aromatic nitrocompounds contain a NO_2_ group and are used in a wide range of therapeutic applications, including antineoplastic activity [52–54]. The biological activity of nitrocompounds appears to involve three important steps: passage through biological membranes, cellular metabolism, and reactivity with biomolecules. Regarding the first step, hydrophobicity is a characteristic shared by the four TSC compounds studied here. Inside cells, the mechanism of action was influenced by the different bulky substituents present at the R2 group. In the case of TSC-NO_2_, the predominance of apoptosis associated with GSH depletion even at lower concentrations of the compound is consistent with enzymatic bioreduction of the NO_2_ group that generate R-NO_2_^•^ [55–57]. The fate of R-NO_2_^•^ is dependent on the oxygenation state of the cells. In aerobic conditions, the reversion of R-NO_2_^•^ to R-NO_2_ by molecular oxygen characterizes a futile cycle, with the generation of the superoxide ion. In anaerobic conditions, the complete reduction to the amine derivative involves the formation of different intermediates, including free radicals and compounds that interact with DNA and are acceptors for thiol groups. Therefore, nitrocompounds are frequently described as efficient against solid tumors, in which hypoxia is commonly present. However, in the present study, TSC-NO_2_ was efficient in aerobic conditions which is consistent with the formation of superoxide ions in the futile cycle leading to GSH depletion. The regeneration of TSC-NO_2_ in the futile cycle is consistent with the depletion of GSH by low concentrations of TSC-NO_2_. Another probable mechanism that operates in aerobic conditions is the alkylation of GSH, as described for nitrocompounds [45]. In this regard, this mechanism could operate for the other studied TSC compounds that is consistent with significant GSH depletion only at concentrations ≥ 50 *μ*M. The present study provided considerable insight into the mechanisms by which these TSC-derivatives exert their effects, and these investigations should be continued in future specific and detailed studies.

## 4. Conclusion

The new TSC compounds presented here exhibit an innovative structure, with benzyl as the R3 group, phenyl as the R2 group, and methyl as the R1 group. This structure endows high hydrophobicity to the molecules and favors transport through membranes. The activity of these compounds against K562 cells was modulated by the different bulky substituents appended to the R2 group. The presence of the group appended to the R2 position was crucial for the induction of cell death by the compounds. All of the TSC compounds studied here promoted the disruption of the mitochondrial potential prior to the death of K562 cells. Apoptosis was favored for compounds with a lower log⁡⁡*P* value and compounds that were unable to chelate iron. Exclusively for TSC-NO_2_, cell death was associated with metabolic effects related to GSH depletion, even at the lowest concentration. Therefore, the effects of those TSC compounds were modulated by the R2 group, leading to different metabolic routes in the cells. The significantly lower toxic effects for PBMNs indicate that these compounds have potential for use as therapeutic agents.

## Figures and Tables

**Table 1:** Calculated and experimental mass compounds obtained by high-resolution mass spectrometry.

Code	Molecular formula	Calculated	Experimental	Error (ppm)^a^
**3a**(TSC-H)	C_16_H_18_N_3_S^+^	284.1216	284.1218	0.70
**3b**(TSC-F)	C_16_H_17_FN_3_S^+^	302.1122	302.1120	−0.66
**3c**(TSC-Cl)	C_16_H_17_ClN_3_S^+^	318.0826	318.0819	−2.20
**3d**(TSC-Me)	C_17_H_20_N_3_S^+^	298.1372	298.1369	−1.01
**3e**(TSC-NO_2_)	C_16_H_17_N_4_O_2_S^+^	329.1067	329.1071	1.22

^a^Acceptable mass errors ≤±5.00 ppm.
